# Predicting a decrease in left atrial appendage flow velocity using left atrial diameter and CHA_2_DS_2_-VASc score in patients with non-valvular atrial fibrillation

**DOI:** 10.1186/s12872-022-03033-6

**Published:** 2023-04-03

**Authors:** Guangyu Wang, Guangyu Li, Feng Hu, Minhua Zang, Jun Pu

**Affiliations:** Department of Cardiology, School of Medicine, Renji Hospital, Shanghai Jiao Tong University, No.160 Pujian Road, Pudong New District, Shanghai, 200120 China

**Keywords:** Left atrial diameter, CHA_2_DS_2_-VASc score, Left atrial appendage flow velocity, Non-valvular atrial fibrillation

## Abstract

**Background:**

Left atrial (LA) appendage flow velocity (LAAFV) is a classic but invasive predictor of thromboembolic events in patients with atrial fibrillation (AF). We aimed to explore the usefulness of LA diameter (LAD) combined with CHA_2_DS_2_-VASc score, which is easily available and non-invasive, as a novel score for predicting a decrease in LAAFV in non-valvular AF (NVAF).

**Methods:**

In total, 716 consecutive NVAF patients who underwent transesophageal echocardiography were divided into the decreased LAAFV (< 0.4 m/s) and preserved LAAFV (≥ 0.4 m/s) groups.

**Results:**

The decreased LAAFV group had a larger LAD and a higher CHA_2_DS_2_-VASc score than the preserved LAAFV group (*P* < 0.001). Multivariate linear regression indicated that brain natriuretic peptide (BNP) concentration, persistent AF, LAD, and CHA_2_DS_2_-VASc score were remained inversely associated with LAAFV. Moreover, multivariate logistic regression revealed that BNP concentration (odds ratio [OR] 1.003, 95% confidence interval [CI] 1.001–1.005, *P* = 0.003), persistent AF (OR 0.159, 95% CI 0.102–0.247, *P* < 0.001), and LAD (OR 1.098, 95% CI 1.049–1.149, *P* < 0.001) were independent factors for a decrease in LAAFV. A novel score, LAD combined with CHA_2_DS_2_-VASc score, was more accurate for predicting a decrease in LAAFV among NVAF patients (area under the curve was 0.733).

**Conclusion:**

Enlarged LAD was independent risk factor for a decrease in LAAFV among NVAF patients. LAD combined with CHA_2_DS_2_-VASc score enhanced the predictive ability for a decrease in LAAFV among NVAF patients.

## Introduction

Atrial fibrillation (AF) is the most frequent type of cardiac arrhythmia [1–3]. The estimated prevalence rate of AF in adults is 0.77% and an age-adjusted rate of 0.61%, suggesting that approximately 8 million patients in China [4]. Ischemic stroke is one of the most feared complications of AF patients. The incidence of stroke is almost five-fold higher in subjects with AF than in those without [5]. Therefore, it is particularly important to determine the stroke risk in patients with AF at an early stage. The CHA_2_DS_2_-VASc score is the most commonly used AF stroke risk stratification schemes in major guidelines [6, 7]. Studies have shown that the CHA_2_DS_2_-VASc score predicts stroke risk and is associated with left atrial (LA) appendage (LAA) flow velocity (LAAFV) [8, 9]. It can thus be used to predict a decrease in LAAFV.

LA diameter (LAD) enlargement, as measured by transthoracic echocardiography (TTE), is associated with AF occurrence, recurrence, and thromboembolic events [10–12]; thus, LAD enlargement confers a high thromboembolic risk. AF-associated thrombus usually forms in the left atrium and LAA. A decrease in LAAFV, as evaluated by transesophageal echocardiography (TEE), is highly correlated with stroke and thrombus formation in patients with AF [13–18]. Therefore, LAAFV has been identified as an independent predictor of stroke and thrombus formation in people with AF. However, TEE is an invasive test and may not be immediately available in routine clinical practice. Additionally, the parameters influencing LAAFV in patients with AF are limited. In this study, we aimed to explore whether LAD combined with CHA_2_DS_2_-VASc score, which is easily available and non-invasive, could be used as a novel score for a decrease in LAAFV among patients with non-valvular AF (NVAF).

## Methods

### Patient enrollment

In total, 716 consecutive patients with NVAF who underwent TTE and TEE were recruited at Renji hospital from January 2019 to October 2021. Indication for TEE in our study was an assessment of cardiac source of embolism. The exclusion criteria included heart valve-associated AF (moderate/severe mitral stenosis and mechanical prosthetic heart valve), severe liver/renal disorders, hyperthyroidism-related AF, and a history of AF catheter ablation or LAA occlusion. Paroxysmal AF was defined as self-terminating spontaneously within 7 days, whereas persistent AF was defined as recurrent AF that was sustained beyond 7 days or that lasted fewer than 7 days but required drug therapy or electrical cardioversion [7]. This study was performed in accordance with the 1975 Helsinki Declaration and was approved by the regional ethics committee.

### Collection of clinical information

The demographic and clinical information, including age, sex, body mass index (BMI), and past medical history, were collected from the electronic medical records of the hospital information system. Once these data were obtained, the CHA_2_DS_2_-VASc score was calculated based on a point system in which one point was assigned for the presence of each of congestive heart failure, hypertension, age ≥ 65 years, diabetes mellitus, vascular disease, and female sex, and two points were assigned for each of age ≥ 75 years and previous stroke or transient ischemic attack.

### Echocardiography study

TTE was performed in all patients using a CX 50 probe (Philips Medical Systems, Eindhoven, Netherlands) or Vivid E9 probe (GE Healthcare, USA) following the current standards of the European Association of Cardiovascular Imaging [19]. LAD was measured from parasternal long axis view using 2-dimensional echocardiography, and left ventricular ejection fraction (LVEF) was calculated using the Simpson’s biplane formula.

TEE was performed with a 5-MHz multiplane transesophageal transducer connected to an ultrasound system (Vivid E9, GE Healthcare). After achieving local pharyngeal anesthesia with lidocaine spray, the patient was placed in the left lateral position and the transesophageal transducer was inserted into the esophagus. The sample volume was placed at 1 cm away from the LAA orifice. LAAFV was measured using the pulsed Doppler method regardless of heart rhythm during TEE. LAAFV was measured and averaged for five cardiac cycles. According to previous study [13], we divided the patients into the decreased LAAFV group (LAAFV < 0.4 m/s) and the preserved group (LAAFV ≥ 0.4 m/s).

### Measurement of blood parameters

White blood cell (WBC) count; platelet (PLT) count; mean platelet volume (MPV); and hemoglobin (Hb), creatinine (Cr), uric acid (UA), fasting blood glucose (FBG), hemoglobin A1c (HbA1c), free fatty acid (FFA), D-dimer (DD), and brain natriuretic peptide (BNP) concentrations were measured in the clinical laboratory at Renji Hospital using the standard laboratory procedures.

### Statistical analysis

Continuous data are presented as means ± standard deviation for normally distributed data or as median (interquartile range) for non-normally distributed data. Categorical data are presented as numbers (percentages). Continuous data were compared using the independent-samples *t*-test, Mann–Whitney U test, or one way analysis or variance, while the chi-square test was used to compare categorical data. Pearson’s and Spearman’s correlation coefficients (r) were used to examine the relationship between LAAFV and other variables. A multivariate linear regression analysis was performed to identify the risk factors for LAAFV. Univariate and multivariate logistic regression analyses were performed to identify the factors associated with a decrease in LAAFV. Receiver operator characteristic (ROC) curves were constructed to test the accuracy of the different risk factors in predicting a decrease in LAAFV, and the Z statistic was constructed to compare the difference in the area under the ROC curve (AUC). The Z-test was performed using MedCalc version 19.0. All other statistical analyses were performed using SPSS version 22.0 for Windows (IBM Corp., Armonk, NY, USA). A two-sided *P* value of < 0.05 was considered statistically significant.

## Results

### Baseline characteristics

The clinical characteristics of the 716 patients are summarized in Table 1. Patients in the decreased LAAFV group were older (*P* < 0.001); had a higher prevalence of persistent AF, hypertension, and ischemic stroke; had higher Cr, UA, HbA1c, BNP, and MPV values; and had a lower PLT count and LVEF. The decreased LAAFV group had a larger LAD and a higher CHA_2_DS_2_-VASc score. No difference was found in other clinical and laboratory data between the two groups (*P* > 0.05).

**Table 1:** Baseline clinical data of all patients

Variables	Decreased group (n = 354)	Preserved group (n = 362)	*P*
*Clinical characters*			
Age (years)	68.1 ± 7.5	63.7 ± 9.8	**< 0.001**
Age ≥ 65 and < 75, n (%)	177 (50.0)	169 (46.7)	0.374
Age ≥ 75, n (%)	64 (18.1)	33 (9.1)	< 0.001
male sex, n (%)	220 (62.1)	215 (59.4)	0.450
BMI (kg/m^2^)	24.77 ± 3.88	24.86 ± 3.41	0.761
Persistent AF, n (%)	258 (72.9)	65 (18)	**< 0.001**
Ischemic stroke, n (%)	111 (31.4)	56 (15.5)	**< 0.001**
Hypertension, n (%)	241 (68.1)	209 (57.7)	**0.004**
CHF, n (%)	112 (31.6)	98 (27.1)	0.272
Vascular disease, n (%)	36 (10.2)	37 (10.2)	0.982
DM, n (%)	78 (22)	61 (16.9)	0.080
Smoker, n (%)	41 (11.6)	46 (12.7)	0.645
CHA_2_DS_2_-VASc score	4 (2–5)	2 (1–3)	**< 0.001**
*Laboratory data*			
WBC (x 10^9^/L)	6.09 ± 1.42	6.16 ± 1.46	0.510
HGB (g/L)	140.36 ± 16.21	139.33 ± 16.42	0.399
PLT (x 10^9^/L)	195.28 ± 52.94	210.07 ± 54.95	**< 0.001**
MPV (fL)	11.22 ± 1.06	10.97 ± 1.11	**0.002**
FBG (mmol/L)	5.06 (4.59–5.77)	4.95 (4.55–5.54)	0.167
HbA1c (%)	5.8 (5.5–6.2)	5.6 (5.4–6.1)	**< 0.001**
Cr (µmol/L)	70 (60–82)	67 (57–80)	**0.004**
UA (µmol/L)	367 (299–435)	350 (296–416)	**0.033**
BNP (pg/mL)	226.9 ± 217.2	84.7 ± 103.4	**< 0.001**
FFA (mmol/L)	0.65 ± 0.32	0.69 ± 0.31	0.140
DD (ug/mL)	0.18 ± 0.17	0.22 ± 1.04	0.554
*Echocardiographic data*			
LAD (mm)	46.3 ± 4.9	41.1 ± 5.3	**< 0.001**
LVEF (%)	61 (55–65)	64 (60–67)	**< 0.001**
LAAFV (m/s)	0.27 (0.22–0.33)	0.60 (0.48–0.78)	**< 0.001**

The bold fonts mean *p* value which has statistical significance

*LAAFV* Left atrial appendage flow velocity, *BMI* Body mass index, *AF* Atrial fibrillation, *CHF* Congestive heart failure, *DM* Diabetes mellitus, *WBC* White blood cell, *Hb* Hemoglobin, *PLT* Platelet, *MPV* Mean platelet volume, *Cr* Creatinine, *UA* Uric acid, *FBG* Fasting blood glucose, *HbA1c* Hemoglobin A1c, *BNP* Brain natriuretic peptide, *FFA* Free fatty acid, *DD* D-dimer, *LAD* Left atrial diameter, *LVEF* Left ventricular ejection fraction

### Relationship between LAAFV and other variables

**Fig. 1:**
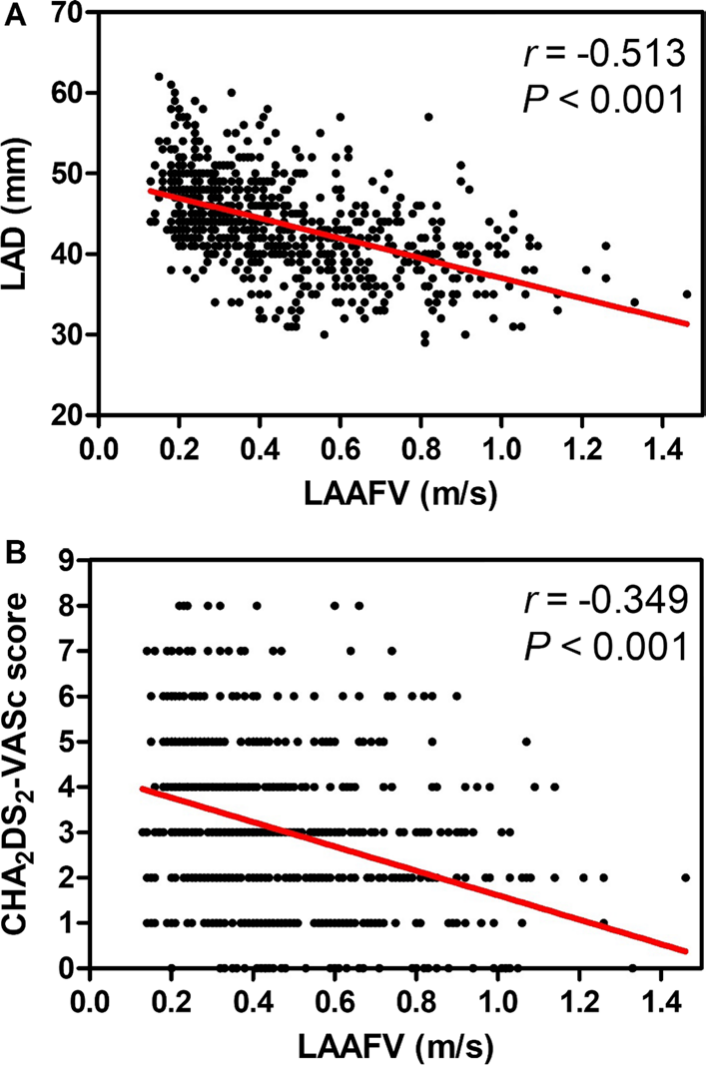
Relationship between LAAFV and LAD (**A**) and CHA2DS2-VASc score (**B**). *LAD* Left atrial diameter, *LAAFV* Left atrial appendage flow velocity, *r* Correlation coefficients

We used Pearson’s and Spearman’s correlation coefficients to identify the continuous and categorical variables that influence LAAFV, respectively. As shown in Table 2 and Fig. 1, LAAFV was related to age, Cr concentration, UA concentration, HbA1c, BNP concentration, PLT count, MPV, CHA_2_DS_2_-VASc score, LAD, and LVEF. Furthermore, LAAFV was associated with persistent AF, hypertension, and ischemic stroke. A multivariate logistic regression analysis was also performed (Table 2). The results show that BNP, CHA_2_DS_2_-VASc score, LAD, and persistent AF were remained markedly associated with LAAFV.

**Table 2:** Relationship between LAAFV and other variables

Variables	Univariate analysis		Multivariate analysis	
	*r*	*P*	Beta	*P*
Age (years)	− 0.217	< 0.001	− 0.059	0.136
Persistent AF	− 0.623	< 0.001	− 0.360	**< 0.001**
Hypertension	− 0.110	0.003	0.037	0.286
Ischemic stroke	− 0.216	< 0.001	− 0.002	0.967
CHA_2_DS_2_-VASc	− 0.349	< 0.001	− 0.134	**0.034**
PLT	0.126	0.001	0.043	0.174
MPV	− 0.146	< 0.001	− 0.035	0.264
BNP	− 0.426	< 0.001	− 0.143	**< 0.001**
Cr	− 0.118	0.002	− 0.004	0.890
UA	− 0.113	0.003	− 0.009	0.787
HbA1c	− 0.127	0.001	− 0.039	0.204
LAD	− 0.513	< 0.001	− 0.227	**< 0.001**
LVEF	0.261	< 0.001	0.031	0.330

The bold fonts mean *p* value which has statistical significance

### Univariate and multivariate analyses to identify factors associated with the decrease in LAAFV

The univariate and multivariate analyses to identify factors associated with the decrease in LAAFV in the overall cohort are shown in Table 3. In the multivariate analysis, age (OR 1.044, 95% CI 1.013–1.075, *P* = 0.005), BNP concentration (OR 1.003, 95% CI 1.001–1.005, *P* = 0.003), LAD (OR 1.098, 95% CI 1.049–1.149, *P* < 0.001), and persistent AF (OR 0.159, 95% CI 0.102–0.247, *P* < 0.001) were independent factors associated with the decrease in LAAFV in patients with NVAF.

**Table 3:** Univariate and multivariate analyses of factors associated with the decrease in LAAFV in all patients

Variables	Univariate analysis			Multivariate analysis		
	OR	95% CI	*P*	OR	95% CI	*P*
Age (years)	1.060	1.041–1.080	< 0.001	1.044	1.013–1.075	**0.005**
Persistent AF	0.081	0.057–0.116	< 0.001	0.159	0.102–0.247	**< 0.001**
Hypertension	0.640	0.472–0.869	0.004	1.060	0.661–1.701	0.809
CHA_2_DS_2_-VASc	1.469	1.340–1.611	< 0.001	1.100	0.933–1.297	0.256
PLT	0.995	0.992–0.998	< 0.001	0.996	0.992-1.000	0.085
MPV	1.241	1.082–1.424	0.002	1.036	0.849–1.266	0.725
BNP	1.009	1.007–1.011	< 0.001	1.003	1.001–1.005	**0.003**
Cr	1.014	1.006–1.022	0.001	1.004	0.992–1.016	0.500
UA	1.002	1.000–1.003	0.020	0.999	0.997–1.002	0.469
HbA1c	1.313	1.086–1.586	0.005	1.150	0.895–1.477	0.275
LAD	1.227	1.183–1.273	< 0.001	1.098	1.049–1.149	**< 0.001**
LVEF	0.926	0.905–0.947	< 0.001	0.972	0.943–1.001	0.055

The bold fonts mean *p* value which has statistical significance

*OR* Odds ratio, *CI* Confidence interval. All other abbreviations are as listed in the footnote of Table 1

### Subgroup analyses by LAD and CHA_2_DS_2_-VASc score

**Fig. 2:**
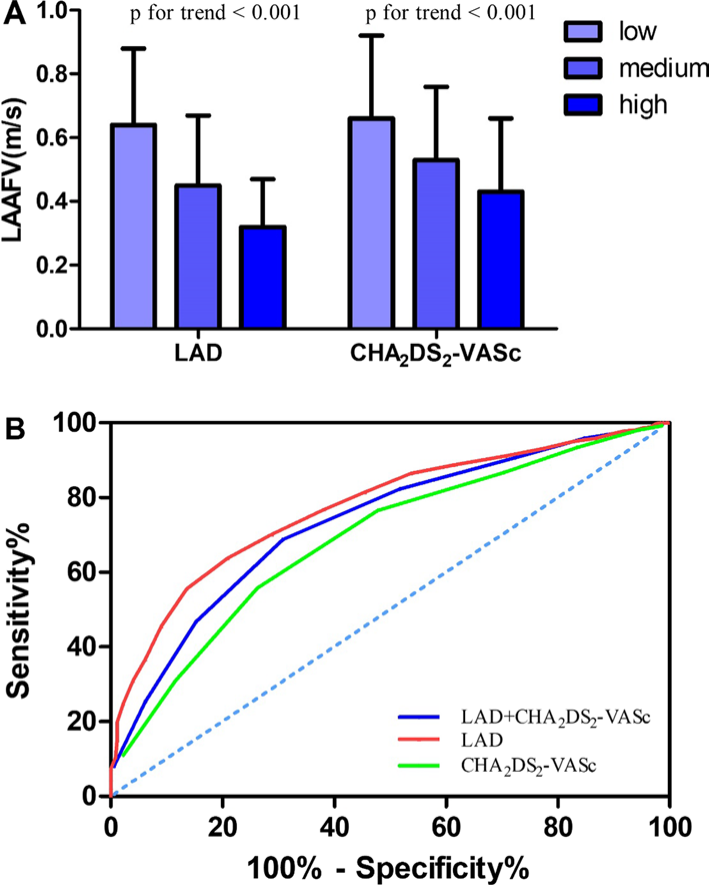
** A** Subgroup analyses by LAD and CHA_2_DS_2_-VASc score. **B** The predictive value of risk factors. *LAD* Left atrial diameter, *LAAFV* Left atrial appendage flow velocity

The subgroup analyses results are shown in Fig. 2A Patients were further stratified into tertiles according to LAD (≤ 41 mm, 42–45 mm, and ≥ 46 mm). The results showed that the LAAFV decreased significantly with an increased in LAD (*P* < 0.001). Furthermore, subgroup analyses were stratified according to the CHA_2_DS_2_-VASc score (low risk = 0, medium risk = 1, and high risk = ≥ 2). The results showed that the LAAFV decreased gradually with an increase in CHA_2_DS_2_-VASc score (*P* < 0.001).

### ROC curve analysis of the decrease in LAAFV

The ROC curve analysis is presented in Table 4; Fig. 2B. One additional point was in the condition that LAD was larger than its optimal cutoff value. The ROC curve analysis demonstrated that the cutoff value for LAD was 42.5 mm (sensitivity: 79%, specificity: 61%, positive predictive value [PPV]: 68%, negative predictive value [NPV]: 73%, AUC: 0.774, 95% CI 0.740–0.808, *P* < 0.001), the cutoff value of CHA_2_DS_2_-VASc score was 2.5 points (sensitivity: 74%, specificity: 56%, PPV: 62%, NPV 68%: AUC: 0.689, 95% CI 0.651–0.728, *P* < 0.001). Then we calculated the total score for LAD + CHA_2_S_2_-VASc score. After the analysis of ROC, we found the cutoff value of LAD combined with the CHA_2_DS_2_-VASc score was 4 points (sensitivity: 70%, specificity: 69%, PPV: 68%, NPV: 72%, AUC: 0.733, 95% CI 0.697–0.770, *P* < 0.001) in predicting the occurrence of a decrease in LAAFV.

**Table 4:** ROC curve analysis of risk factors

Risk factors	AUC	Cutoff	Sensitivity (%)	Specificity (%)	PPV (%)	NPV (%)	95% CI	*P*
LAD	0.774	42.5	79	61	68	73	0.740–0.808	**< 0.001**
CHA_2_DS_2_-VASc	0.689	2.5	74	56	62	68	0.651–0.728	**< 0.001**
LAD + CHA_2_DS_2_-VASc	0.733	4.0	70	69	68	72	0.697–0.770	**< 0.001**

The bold fonts mean *p* value which has statistical significance

*AUC* Area under curve, *CI* Confidence interval. *PPV* Positive predictive value. *NPV* Negative predictive value. All other abbreviations are as listed in the footnote of Table 1

MedCalc software was used to compare the various ROC curves, and the results are shown in Table 5. The AUC of LAD + CHA_2_DS_2_-VASc score was significantly larger than that of the CHA_2_DS_2_-VASc score (*P* < 0.0001). Although the AUC of LAD + CHA_2_DS_2_-VASc score was less than that of LAD alone (*P* = 0.0427), the specificity of the combined model was improved (Table 4). Therefore, the combined use of LAD + CHA_2_DS_2_-VASc score could significantly improve the ability of these parameters to predict a decrease in LAAFV in patients with NVAF.

**Table 5:** Comparison of different ROC curves

Different ROC curves	Z	*P*
LAD vs. CHA_2_DS_2_-VASc	3.558	**0.0004**
LAD + CHA_2_DS_2_-VASc vs. LAD	2.026	**0.0427**
LAD + CHA_2_DS_2_-VASc vs. CHA_2_DS_2_-VASc	8.329	**< 0.0001**

The bold fonts mean *p* value which has statistical significance

All abbreviations are as listed in the footnote of Table 1

## Discussion

Our study investigated the association among LAD, CHA_2_DS_2_-VASc score, and LAAFV in 716 patients with NVAF. To the best of our knowledge, this is the first study to demonstrate that patients with a larger LAD and a higher CHA_2_DS_2_-VASc score are prone to a decrease in LAAFV. Additionally, the results of the ROC curve analysis showed that the predictive ability of LAD + CHA_2_DS_2_-VASc score in predicting a decrease in LAAFV was better, suggesting that LAD combined with CHA_2_DS_2_-VASc score enhanced the predictive ability for a decrease in LAAFV among NVAF patients.

The LAA is a major thromboembolic source in patients with AF. As such, many studies have assessed the risk of stroke by analyzing LAAFV [13, 15]. A decrease in LAAFV has been well identified as a surrogate for cardioembolic risk in patients with NVAF. Several studies have shown that a low LAAFV is associated with a higher risk of stroke/thromboembolic events than a high LAAFV in patients with AF [13, 15, 17, 18]. Although TEE is a reliable method to evaluate LAAFV, it is relatively invasive and low yield. Furthermore, knowledge on the factors that influence LAAFV is limited. The LAA is adjacent to the left atrium; thus, the LAAFV is susceptible to LA remodeling. A previous study showed a significant negative correlation between LA volume and LAAFV [20]. In addition, a study by Schnieder et al. reported that LAD is inversely correlated with LAAFV [21]. Our study showed that LAD is negatively and linearly correlated with LAAFV, meaning that an increase in LAD parallels to a decrease in LAAFV. Additionally, the multivariate analysis demonstrated that LAD is an independent risk factor for the decrease in LAAFV after adjusting for other variables. For every additional unit change in LAD, the odds of a decrease in LAAFV in patients with AF increased by 1.098 times. In the subgroup analysis, as the LAD increased, the LAAFV decreased (*P* < 0.001). These subgroup analyses further validated the relationship between LAD and LAAFV at different levels. In a previous study on patients with non-valvular paroxysmal AF, LAD was an independent predictor of a decrease in LAAFV in patients with sinus rhythm (SR) during TEE [22]. Another study by Fukuhara et al. found that LA volume index could predict a decrease in LAAFV during SR in patients with AF, but a considerable proportion of patients with AF rhythm were excluded from this study [20]. In our study, we chose the LAD as the study target because it is practical and easy to obtain clinically. Furthermore, LA volume index was not used for the routine measure of LA enlargement in our hospital. Notably, our study provided a specific cutoff value for LAD (42.5 mm) to predict the decrease in LAAFV, which is helpful for clinicians to evaluate of stroke risk in patients with NVAF.

The CHA_2_DS_2_-VASc score has been widely used to predict the risk of ischemic stroke in patients with AF. Recent guidelines recommend anticoagulant therapy in high-risk patients with a CHA_2_DS_2_-VASc score of ≥ 2 [6, 7]. The relationship between stroke/thrombus formation and LAAFV has been investigated in many studies, and an LAAFV of ≤ 0.4 m/s represents a risk of stroke/thrombus [13]. However, the study of relationship between the CHA_2_DS_2_-VASc score and LAAFV is rare. In the present study, the CHA_2_DS_2_-VASc score (beta = − 0.134, *P* = 0.034) was significantly associated with LAAFV according to the multivariate linear regression analysis, while in multivariate logistics analysis CHA_2_DS_2_-VASc is no longer significant. Previous study showed that the CHA2DS2-VASc score was an independent predictor of a decrease in LAAFV [23]. Possible explanations include the variations in the recruitment criteria and the fact that the CHA_2_DS_2_-VASc score served as a categorical variable. Nevertheless, the ROC curve analysis demonstrated that the AUC was 0.689, with a sensitivity of 74% and a specificity of 56% when using the CHA_2_DS_2_-VASc score to predict the decrease in LAAFV in patients with NVAF. The predictive power of the CHA_2_DS_2_-VASc score was modest; thus, we further sought to develop a combined model that might better predict the decrease in LAAFV as a surrogate for cardioembolic risk in patients with NVAF. In the present study, LAD was an independent risk factor for the decrease in LAAFV. Combined use of LAD and CHA_2_DS_2_-VASc score significantly increased the predictive ability for the decrease in LAAFV compared with CHA_2_DS_2_-VASc score alone in patients with NVAF. In fact, LAD has been shown to be an independent risk factor for stroke/thrombus formation in patients with NVAF [24–26]. Therefore, a combination of LAD and CHA_2_DS_2_-VASc score could be used as a substitute to predict the decrease in LAAFV in patients with NVAF.

We also showed that LAAFV is related to BNP concentration, which is in agreement with previous studies showing that BNP concentration is significantly inversely correlated with LAAFV in patients with AF [20]. In this study, we found that the persistent AF decreased the risk of decreased LAAFV in multivariate logistics analysis, which was opposite to the results of the linear analysis and previous study [27]. The paradoxical results were mainly caused by the cutoff value of LAAFV we chose. A variety of cutoff values of LAAFV (0.2 m/s, 0.35 m/s, 0.4 m/s) have been reported in previous studies [17, 20, 23]. Different cutoff values have an impact on the results of subsequent statistical analysis. The mean velocity of LAAFV of all patients in our study was 0.46 m/s. Therefore, we set the cutoff value of LAAFV to 0.4 m/s.

Although a decrease in LAAFV is not indicated for oral anticoagulants following the current guidelines, reduced LAAFV is highly correlated with stroke and thrombus formation in patients with AF. Our study showed that the combined model of LAD and CHA2DS2-VASc score may better predict a decrease in LAAFV, suggesting the new model facilitating thromboembolic risk stratification in AF patients under the routine screening.

## Limitations

The present study had several limitations. First, this was a single- center retrospective study. Thus, we could not get some information in medical records, such as whether the patients were AF rhythm during TEE. Furthermore, our results need to be further confirmed in a multi-center study. Second, there is no uniform criteria for the decrease in LAAFV in patients with AF. A variety of criteria for the decrease in LAAFV have been reported and the method of measuring LAAFV also varies in different studies. Third, the patients in this study were prepared for catheter ablation or LAA occlusion. Therefore, the results may not be fully representative of all patients with NVAF. Finally, though LA volume index is more accurate measure of LA size than LAD, this test is not routinely used for patients in our hospital. Therefore, we chosen easily-obtained LAD in this study.

## Conclusion

In conclusion, on the basis of a relatively large hospital-based sample, our study demonstrated that LAD combined with CHA_2_DS_2_-VASc score, a novel score, showed a better ability to predict the decrease in LAAFV in patients with NVAF. However, our conclusions need further validation in large-sample multi-center studies.

## Data Availability

The dataset of this article is accessible on reasonable from the corresponding author.

## Abbreviations

AF: Atrial fibrillation
NVAF: Non-valvular atrial fibrillation
TTE: Transthoracic echocardiography
TEE: Transesophageal echocardiography
LAAFV: Left atrial appendage flow velocity
BMI: Body mass index
DM: Diabetes mellitus
WBC: White blood cell
Hb: Hemoglobin
PLT: Platelet
MPV: Mean platelet volume
Cr: Creatinine
UA: Uric acid
FBG: Fasting blood glucose
HbA1c: Hemoglobin A1c
BNP: Brain natriuretic peptide
FFA: Free fatty acid
DD: D-dimer
LA: Left atrial
LAD: Left atrial diameter
LVEF: Left ventricular ejection fraction
